# Comparing pre-operative versus post-operative single and multi-fraction stereotactic radiotherapy for patients with resectable brain metastases

**DOI:** 10.1016/j.ctro.2022.11.004

**Published:** 2022-11-09

**Authors:** Haley K. Perlow, Cindy Ho, Jennifer K. Matsui, Rahul N. Prasad, Brett G. Klamer, Joshua Wang, Mark Damante, Rituraj Upadhyay, Evan Thomas, Dukagjin M. Blakaj, Sasha Beyer, Russell Lonser, Douglas Hardesty, Raju R. Raval, Roshan Prabhu, James B. Elder, Joshua D. Palmer

**Affiliations:** a Department of Radiation Oncology, The Ohio State University Wexner Medical Center, Columbus, OH, USA; b Ohio State University School of Medicine, Columbus, OH, USA; c Center for Biostatistics, The Ohio State University, Columbus, OH, USA; d Department of Neurosurgery, The Ohio State University Wexner Medical Center, Columbus, OH, USA; e Levine Cancer Institute, Atrium Health, Charlotte, NC, USA; f Southeast Radiation Oncology Group, Charlotte, NC, USA

**Keywords:** Brain metastases, Radionecrosis, Leptomeningeal disease, Pre-operative, Radiotherapy

## Abstract

**Background:**

The standard treatment for patients with large brain metastases and limited intracranial disease is surgical resection and post-operative stereotactic radiosurgery (SRS). However, post-operative SRS still has elevated rates of local failure (LF) and is complicated by radiation necrosis (RN), and meningeal disease (MD). Pre-operative SRS may reduce the risk of RN and MD, while fractionated therapy may improve local control through delivering a higher biological effective dose. We hypothesize that pre-operative fractionated stereotactic radiation therapy (FSRT) will have less toxicity compared to patients who receive post-operative SRS or FSRT.

**Methods:**

A multi-institutional analysis was conducted and included patients who had surgical resection and stereotactic radiation therapy to treat at least one brain metastasis. Pertinent demographic, clinical, radiation, surgical, and follow up data were collected for each patient. The primary outcome was a composite endpoint defined as patients with one of the following adverse events: 1) LF, 2) MD, and/or 3) Grade 2 or higher (symptomatic) RN.

**Results:**

279 patients were eligible for analysis. The median follow-up time was 9 months. 87 % of patients received fractionated treatment. 29 % of patients received pre-operative treatment. The composite endpoint incidences for post-operative SRS (n = 10), post-operative FSRT (n = 189), pre-operative SRS (n = 27), and pre-operative FSRT (n = 53) were 0 %, 17 %, 15 %, and 7.5 %, respectively.

**Conclusions:**

In our study, the composite endpoint of 7.5% for pre-operative FSRT compares favorably to our post-operative FSRT rate of 17%. Pre-operative FSRT was observed to have low rates of LF, MD, and RN. Prospective validation is needed.

## Highlights

• Pre-operative radiation therapy for brain metastases may reduce meningeal disease.
• Pre-operative radiation therapy for brain metastases may reduce radionecrosis.
• Fractionated radiation therapy for brain metastases may reduce local failure.
• Fractionated pre-operative radiation therapy requires prospective validation.

## Introduction

Brain metastases are a cause of substantial morbidity and mortality for cancer patients. Whole brain radiation therapy (WBRT) plus surgery is a historical standard that has been shown to improve overall survival (OS) and local control for patients with brain metastases [1]. Adjuvant WBRT after surgical resection of a brain metastasis, compared to surgery alone, has been shown to reduce the risk of intracranial recurrence and neurological death, although without improving OS [2]. Unfortunately, multiple randomized studies have shown significant neurological and cognitive toxicity with WBRT [3], [4]. Multiple studies have shown how post-operative stereotactic radiosurgery (SRS) is an acceptable alternative to WBRT that decreases the risk of cognitive dysfunction without negatively affecting overall survival (OS) [5], [6]. However, post-operative SRS may increase the incidence of local failure (LF), radiation necrosis (RN), and meningeal disease (MD).

It has been speculated that pre-operative radiation therapy may decrease the risk of these adverse outcomes when compared to post-operative SRS. Less brain tissue is treated when radiating brain metastases prior to surgery which may minimize the risk of RN. Pre-operative SRS may reduce the risk of tumor spillage and subsequent MD which is often seen when radiation was delivered after surgery. Also, up to 30 % of patients do not receive their prescribed course of post-operative radiation therapy due to treatment non-compliance or medical complications [6]. The PROPS-BM cohort retrospectively evaluated outcomes for patients treated with pre-operative SRS; however, even though they showed a minimized risk of RN and MD, the one year incidence of LF rate remained elevated at 15 % [7]. Fractionation may facilitate safer delivery of higher biological effective dose (BED) treatments which may improve the high LF rates seen in N107C, Mahajan et al, and the PROPS-BM cohort [5], [7], [8]. This has been shown in a previously published cohort that did not show any incidences of LF in patients who received pre-operative fractionated stereotactic radiation therapy (FSRT) [9]. Currently, Alliance A071801 is comparing post-operative SRS with post-operative FSRT, and NRG BN012 is comparing pre-operative SRS with post-operative SRS. However, a direct comparison between pre-operative and post-operative SRS and FSRT has not been previously published. We hypothesize that our novel cohort of patients who received pre-operative FSRT will have a lower combined rate of LF, RN, and MD when directly compared to a large cohort of patients who received post-operative FSRT.

## Methods

This retrospective study was approved by our institutional review board. Data was pooled from two institutions for analysis. Patients who had surgical resection of at least 1 metastasis and a pre- or post-operative stereotactic radiation course delivered to at least 1 brain metastasis or surgical cavity were retrospectively identified. Patients with more than one lesion removed at the time of surgery were included. Patients who had single or multi-fraction treatment were eligible for inclusion. While postoperative radiation therapy is a standard-of-care for brain metastases, at both institutions, patients were eligible for pre-operative fractionated stereotactic radiation therapy (FSRT) if they had a new dominant brain metastasis, limited intracranial disease, a good overall prognosis, at the discretion of the treating neurosurgeon and radiation oncologist. At both institutions, pre-operative FSRT was scheduled 1–2 weeks after radiation oncology and neurosurgery evaluation for a diagnosis of new or progressive brain metastases, with surgery typically performed on the same day or within a week from the last radiation treatment. If radiation therapy was delivered post-operatively, it typically started roughly 4 weeks after surgical resection.

Pertinent demographic, clinical, radiation, surgical, and follow up data were collected for each patient. Patients were defined as having uncontrolled extracranial disease if they had progressive extracranial disease or were treatment naïve at the time of their brain metastasis diagnosis. A patient was defined as having absent extracranial metastases if there was no extracranial disease outside of the primary tumor and regional lymph nodes at the time of their brain metastasis diagnosis. Karnofsky performance status (KPS) was documented at the time of radiation oncology consultation. Radionecrosis (RN) was defined as any radiographic post-treatment change felt by a multidisciplinary team (radiation oncology, neurosurgery, neuro-oncology, neuro-radiology) to be consistent with treatment effect rather than disease progression. RN was graded according to the NCI Common Terminology Criteria for Adverse Events (CTCAE) v5.0 (Grade 1: asymptomatic; Grade 2: moderate symptoms, corticosteroids indicated; Grade 3: severe symptoms, medical intervention indicated; Grade 4: life-threatening; urgent intervention; and Grade 5: death). LF was defined as radiographic changes determined by a multidisciplinary team to be suspicious for progression or pathologically proven disease progression. MD was defined as radiographic evidence as per multidisciplinary team. A composite endpoint was measured for each patient; this composite endpoint was defined as patients with either: 1) LF, 2) MD, or 3) Grade 2 or higher (symptomatic) RN. This composite endpoint is similar to the composite endpoint for NRG- BN012 which compares pre- and post-operative SRS and is currently enrolling patients. A patient was positive for this composite endpoint if they met any of these three criteria.

Patients were simulated for radiation therapy in the supine position with either a thermoplastic mask or headframe for immobilization. T1 post-contrast volumetric MRI imaging was fused for gross tumor volume (GTV) delineation. For pre-operative treatment, the GTV was defined as the contrast enhanced tumor and adjacent abutting meninges. For post-operative treatment, the GTV was defined as the surgical cavity, any residual enhancing tumor, and adjacent abutting meninges. Radiation therapy was delivered using either a linear accelerator or Gamma Knife radiosurgery platform. For both pre- and post-operative treatments linear accelerator-based treatments, a clinical treatment volume (CTV) of 2 mm and an optional 1 mm planning treatment volume (PTV) were used for all lesions. Linear-accelerator based radiation therapy was planned and delivered uniformly using intensity modulated radiation therapy (IMRT) or volumetric modulated arc therapy-based planning with 2–3 non-coplanar 6 megavoltage arcs with daily image guidance using cone beam CT. Gamma Knife-based SRS was prescribed to the 50 % isodose line. Patients were followed with a detailed history and physical and brain MRI every 2–3 months for the first year, 3–4 months for the second year, and every 6 months after 2 years.

## Statistics

Descriptive statistics were used to summarize baseline demographic, clinical, and treatment variables. The median and interquartile range (IQR) were used for continuous variables and frequency counts and proportions for categorical variables. Patients were stratified into treatment groups by sequencing of therapy (pre-operative vs post-operative radiation therapy) and fractionation (SRS versus FSRT). Differences between groups were summarized using Wilcoxon’s rank sum test or Fisher’s exact test. Overall survival was defined as the date of onset of treatment to the date of death and censored at the date of last follow up for those still alive. The log-rank test and Kaplan-Meier estimate of survival with 95 % confidence interval (CI) were used to compare overall survival outcomes between treatment strategy groups. Gray’s test was used to compare the cumulative incidence of the composite endpoint between treatment strategy groups with death as a competing risk. Cox regression models, adjusting for patient sex, age, and treatment strategy, were used for exploratory analysis of association between overall survival and selected covariates. Complete case analysis was used for all summaries and reported p-values and CIs were unadjusted for multiplicity. Statistical analyses were performed with R version 4.1.2 using the survival (version 3.2–13) and cmprsk (version 2.2–11) packages.

## Results

Between the dates of 1/1/2016 and 12/31/2020, 279 patients with surgical resection for brain metastases were identified and eligible for analysis. The median follow-up time through last follow-up or time of death was 9 months (IQR: 4, 19). During the follow up period, 138 deaths (49 %) were observed. 29 % of patients received pre-operative radiation therapy. (Table 1). A plurality of patients (45 %) had metastatic lung cancer, with genitourinary (16 %), melanoma (13 %) and breast (9.3 %) forming a smaller proportion of patients. More than half (55 %) of patients had extracranial disease control at the time of their radiation treatment, and 48 % of patients had extracranial metastases present during treatment. 88 % of patients had a KPS of ≥ 70 at the time of their radiation oncology evaluation.

**Table 1:** Patient Characteristics.

Characteristic	Postoperative FSRT, N = 189^1^	Preoperative FSRT, N = 53^1^	Postoperative SRS, N = 10^1^	Preoperative SRS, N = 27^1^	p-value^2^	Overall, N = 279^1^
**Sex**					0.3	
Male	116 (61 %)	29 (55 %)	5 (50 %)	12 (44 %)		162 (58 %)
Female	73 (39 %)	24 (45 %)	5 (50 %)	15 (56 %)		117 (42 %)
**Age (years)**	62 (55, 70)	62 (54, 72)	66 (52, 68)	61 (54, 66)	0.8	62 (54, 70)
**Primary Tumor**					—	
Lung	75 (40 %)	27 (51 %)	6 (60 %)	18 (67 %)		126 (45 %)
GU	29 (15 %)	9 (17 %)	2 (20 %)	5 (19 %)		45 (16 %)
Melanoma	29 (15 %)	4 (7.5 %)	0 (0 %)	3 (11 %)		36 (13 %)
Breast	20 (11 %)	6 (11 %)	0 (0 %)	0 (0 %)		26 (9.3 %)
GI	11 (5.8 %)	3 (5.7 %)	2 (20 %)	0 (0 %)		16 (5.7 %)
Head and Neck	14 (7.4 %)	1 (1.9 %)	0 (0 %)	0 (0 %)		15 (5.4 %)
Sarcoma	4 (2.1 %)	2 (3.8 %)	0 (0 %)	0 (0 %)		6 (2.2 %)
GYN	5 (2.6 %)	0 (0 %)	0 (0 %)	1 (3.7 %)		6 (2.2 %)
Other	2 (1.1 %)	1 (1.9 %)	0 (0 %)	0 (0 %)		3 (1.1 %)
**Extracranial Disease Control**					0.13	
Controlled	105 (56 %)	23 (43 %)	6 (60 %)	19 (70 %)		153 (55 %)
Uncontrolled	84 (44 %)	30 (57 %)	4 (40 %)	8 (30 %)		126 (45 %)
**Extracranial Metastases**					0.6	
Absent	96 (51 %)	29 (55 %)	4 (40 %)	17 (63 %)		146 (52 %)
Present	93 (49 %)	24 (45 %)	6 (60 %)	10 (37 %)		133 (48 %)
**KPS**					0.12	
≤60	22 (12 %)	6 (11 %)	4 (40 %)	1 (3.7 %)		33 (12 %)
70–80	90 (48 %)	31 (58 %)	3 (30 %)	16 (59 %)		140 (50 %)
90–100	77 (41 %)	16 (30 %)	3 (30 %)	10 (37 %)		106 (38 %)
**SIMT**					**0.001**	
No	93 (49 %)	28 (53 %)	1 (10 %)	21 (78 %)		143 (51 %)
Yes	96 (51 %)	25 (47 %)	9 (90 %)	6 (22 %)		136 (49 %)
**Number of Fractions**					—	
1	0 (0 %)	0 (0 %)	10 (100 %)	27 (100 %)		37 (13 %)
3	130 (69 %)	49 (92 %)	0 (0 %)	0 (0 %)		179 (64 %)
5	59 (31 %)	4 (7.5 %)	0 (0 %)	0 (0 %)		63 (23 %)
**GTV Volume (cc)**	17 (8, 28)	12 (7, 19)	9 (5, 13)	3 (2, 6)	**<0.001**	14 (6, 24)
**PTV Volume (cc)**	38 (22, 60)	19 (12, 28)	15 (13, 31)	6 (4, 11)	**<0.001**	27 (13, 48)
**Prior Brain RT**					**0.031**	
No	163 (86 %)	52 (98 %)	8 (80 %)	23 (85 %)		246 (88 %)
Yes	26 (14 %)	1 (1.9 %)	2 (20 %)	4 (15 %)		33 (12 %)
**Number of Resected Lesions**					0.5	
1	166 (88 %)	51 (96 %)	8 (80 %)	26 (96 %)		251 (90 %)
2	20 (11 %)	2 (3.8 %)	2 (20 %)	1 (3.7 %)		25 (9.0 %)
3	2 (1.1 %)	0 (0 %)	0 (0 %)	0 (0 %)		2 (0.7 %)
4	1 (0.5 %)	0 (0 %)	0 (0 %)	0 (0 %)		1 (0.4 %)
**Location of Resected Lesion**					—	
Frontal	65 (34 %)	19 (36 %)	4 (40 %)	14 (52 %)		102 (37 %)
Parietal	40 (21 %)	6 (11 %)	1 (10 %)	4 (15 %)		51 (18 %)
Cerebellar	30 (16 %)	12 (23 %)	2 (20 %)	2 (7.4 %)		46 (16 %)
Occipital	22 (12 %)	11 (21 %)	0 (0 %)	2 (7.4 %)		35 (13 %)
Temporal	18 (9.5 %)	3 (5.7 %)	1 (10 %)	4 (15 %)		26 (9.3 %)
Multiple regions	12 (6.3 %)	2 (3.8 %)	2 (20 %)	1 (3.7 %)		17 (6.1 %)
Other	2 (1.1 %)	0 (0 %)	0 (0 %)	0 (0 %)		2 (0.7 %)
**Extent of Resection**					—	
Gross total resection	189 (100 %)	52 (98 %)	10 (100 %)	27 (100 %)		278 (100 %)
Near resection	0 (0 %)	1 (1.9 %)	0 (0 %)	0 (0 %)		1 (0.4 %)
Subtotal resection	0 (0 %)	0 (0 %)	0 (0 %)	0 (0 %)		0 (0 %)

^1^n (%); Median (IQR).

^2^Fisher's exact test; Kruskal-Wallis rank sum test.

KPS - Karnofsky performance status.

SIMT - single isocenter multitarget.

GTV - gross tumor volume.

CTV - clinical target volume.

ccs - cubic centimeters.

17 patients (6.1 %) had multiple lesions resected (Table 1). Nearly all patients (99.6 %) had a gross total resection. The two most common locations of resected lesions were the frontal lobe (37 %) and parietal (18 %). Patients in this study were either treated with a linear accelerator (n = 270) or with Gamma Knife radiosurgery (n = 9). Almost half (49 %) of patients had single isocenter multi-target (SIMT) radiosurgery, and 14 % had five or more lesions treated during the radiation treatment course. The median GTV volume for pre-operative and post-operative treatment was 9 ccs (IQR: 4, 17) and 17 ccs (IQR: 8, 27), respectively (p < 0.001); the median PTV volume for pre-operative and post-operative treatment was 14 ccs (IQR: 7, 25) and 38 ccs (IQR: 22, 59), respectively (p < 0.001). Overall, the median prescribed radiation dose on the linear accelerator was 24 Gy (range of 14–30 Gy), and 18–20 Gy was prescribed to the 50 % isodose line for patients treated with Gamma Knife (all Gamma Knife treatments were single fraction). 13 % of patients were prescribed 1 fraction, 64 % of patients were prescribed 3 fractions, and 23 % of patients were prescribed 5 fractions. All fractionated treatments were delivered on a daily schedule. For pre-operative treatments, the median time from last radiation fraction to surgery was 2 days (IQR: 1, 4.5) and only 3 patients had their surgery more than one week after the last radiation treatment.

**Fig. 1:**
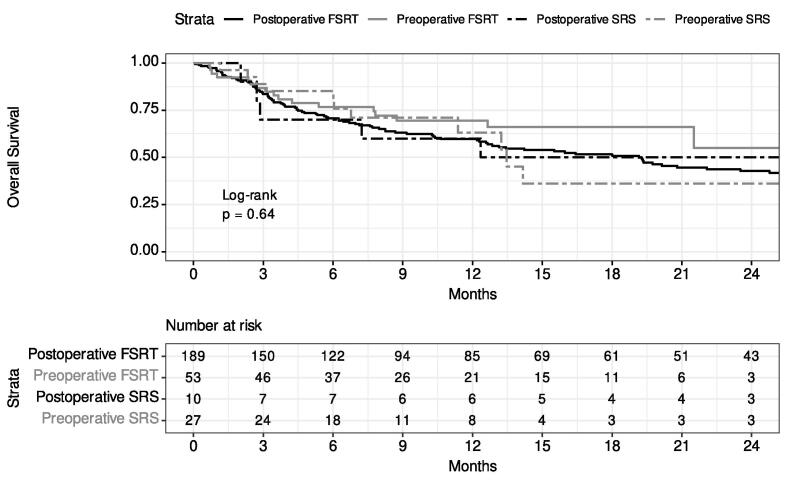
Kaplan-Meier estimate of overall survival stratified by treatment strategy. OS – overall survival. FSRT – fractionated stereotactic radiation therapy, SRS – stereotactic radiosurgery.

The 12-month overall survival probability was 0.62 (95 % CI: 0.56, 0.68). There was no significant difference in survival between the treatment strategy groups (p = 0.64) (Fig. 1). Patients with extracranial metastases present at time of their radiation course had worse overall survival (HR: 2.10; 95 % CI: 1.48, 2.96, p < 0.001). Worse overall survival was also found for patients with lower KPS (≤60 vs 90–100 HR: 4.9; 95 % CI: 2.61, 9.11; and 70–80 vs 90–100 HR: 1.9; 95 % CI: 1.15, 3.01).

Overall, 15 % of patients were positive for the composite endpoint. (Table 2). 7.9 % of patients experienced Grade 2 or higher (G2 + ) RN. There were no Grade 4 or 5 RN events. 4.3 % of patients had MD; 67 % were classical leptomeningeal disease, and 33 % were nodular MD. 3.2 % of patients experienced LF. The rates of LF, G2 + RN, and MD with postoperative FSRT (n = 189) were 4.2 %, 9 %, and 5.3 %, respectively, for a composite endpoint incidence of 17 %. The rates of LF, G2 + RN, and MD with preoperative FSRT (n = 53) were 0 %, 5.7 %, and 1.9 %, respectively, for a composite endpoint incidence of 7.5 %. The rates of LF, G2 + RN, and MD with postoperative SRS (n = 10) were all 0 % for a composite endpoint incidence of 0 %. The rates of LF, G2 + RN, and MD with preoperative SRS (n = 27) were 3.7 %, 7.4 %, and 3.7 %, respectively, for a composite endpoint incidence of 15 %.

**Table 2:** Patient Outcomes.

Characteristic	Postoperative FSRT, N = 189^1^	Preoperative FSRT, N = 53^1^	Postoperative SRS, N = 10^1^	Preoperative SRS, N = 27^1^	p-value^2^	Overall, N = 279^1^
**Local Failure**					0.5	
No	181 (96 %)	53 (100 %)	10 (100 %)	26 (96 %)		270 (97 %)
Yes	8 (4.2 %)	0 (0 %)	0 (0 %)	1 (3.7 %)		9 (3.2 %)
**Radiation Necrosis Grade**					0.8	
0/1	172 (91 %)	50 (94 %)	10 (100 %)	25 (93 %)		257 (92 %)
2	7 (3.7 %)	1 (1.9 %)	0 (0 %)	2 (7.4 %)		10 (3.6 %)
3	10 (5.3 %)	2 (3.8 %)	0 (0 %)	0 (0 %)		12 (4.3 %)
4	0 (0 %)	0 (0 %)	0 (0 %)	0 (0 %)		0 (0 %)
5	0 (0 %)	0 (0 %)	0 (0 %)	0 (0 %)		0 (0 %)
**Leptomeningeal Disease**					0.9	
No	179 (95 %)	52 (98 %)	10 (100 %)	26 (96 %)		267 (96 %)
Yes	10 (5.3 %)	1 (1.9 %)	0 (0 %)	1 (3.7 %)		12 (4.3 %)
**Type of Leptomeningeal Disease**					0.6	
Classic	7 (70 %)	0 (0 %)	0 (NA%)	1 (100 %)		8 (67 %)
Nodular	3 (30 %)	1 (100 %)	0 (NA%)	0 (0 %)		4 (33 %)
Both	0 (0 %)	0 (0 %)	0 (NA%)	0 (0 %)		0 (0 %)
Disease free	179	52	10	26		267
**Composite Endpoint**					0.22	
Negative	156 (83 %)	49 (92 %)	10 (100 %)	23 (85 %)		238 (85 %)
Positive	33 (17 %)	4 (7.5 %)	0 (0 %)	4 (15 %)		41 (15 %)
**Median Follow Up (months)**	9 (4, 23)	9 (4, 16)	15 (4, 31)	7 (5, 13)	0.7	9 (4, 19)
**Mortality**					0.64	
Alive	87 (46 %)	35 (66 %)	3 (30 %)	16 (59 %)		141 (51 %)
Dead	102 (54 %)	18 (34 %)	7 (70 %)	11 (41 %)		138 (49 %)

Three patients (6 %) who received pre-operative FSRT experienced serious post-surgical complications. One patient had an acute right subdural hemorrhage after surgery and was brought back to the operating room for evacuation. A second patient experienced a Methicillin-resistant Staphylococcus Aureus (MRSA) infection post-operatively. Another patient had a clot in the resection cavity-five days after surgery and developed hydrocephalus requiring clot removal in the operating room.

**Fig. 2:**
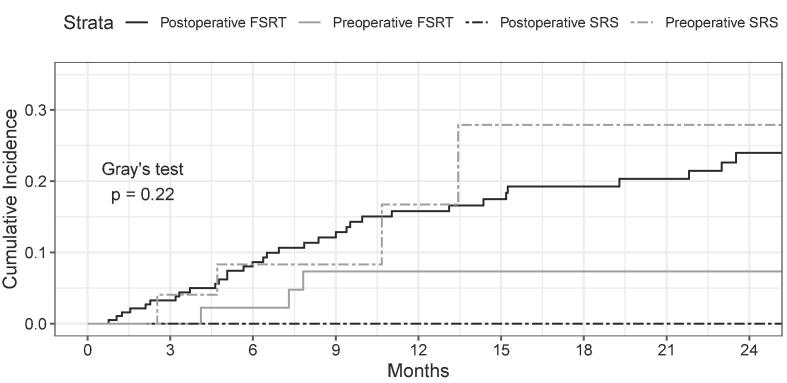
Cumulative incidence curves for the composite endpoint treating death as a competing event. FSRT – fractionated stereotactic radiation therapy, SRS – stereotactic radiosurgery.

Of the 41 patients who were positive for the composite endpoint, 25 (61 %) were deceased at last follow up. The cumulative incidence for experiencing the composite endpoint, stratified by treatment strategy, with death as a competing event is illustrated in Fig. 2. At 12 months, the cumulative probabilities of the composite endpoint for postoperative FSRT, preoperative FSRT, postoperative SRS, and preoperative SRS were 0.16 (95 % CI: 0.11, 0.22), 0.074 (95 % CI: 0.018, 0.18), 0.0 (95 % CI: N/A), and 0.17 (95 % CI: 0.031, 0.40), respectively.

## Discussion

This is the largest known study comparing LF, MD, and RN outcomes between pre- and post-operative FSRT. Our analysis suggests that pre-operative fractionated therapy is a well-tolerated and effective alternative to postoperative SRS or FSRT with an objectively low rate of LF, MD, and RN and without a delay in time to surgery or elevated rate of surgical complications. There were no incidences of LF with pre-operative FSRT with just 3 patients (6 %) experiencing symptomatic RN and 1 patient (2 %) experiencing MD. These data support that pre-operative FSRT should be evaluated prospectively.

After resection of brain metastases, adjuvant radiation therapy is indicated to decrease the risk of local failure[1], [2], [8]. Multiple trials have established that SRS is an acceptable alternative to WBRT that decreases the risk of cognitive dysfunction without impacting OS [5], [6]. Because postoperative SRS is best studied prospectively [5], [6], [8], it is considered to be standard of care [10]. However, suboptimal rates of RN, MD, and 1-year LF of 0–3 %, 7–28 %, and roughly 25–50 % respectively were observed with postoperative SRS in modern, phase III trials [5], [6], [8]. A phase II trial of postoperative SRS for brain metastases noted a lower 1-year rate of LF of 15 % but demonstrated a pathologically-proven RN of 18 %[11]. This better reflects the elevated rates of RN after postoperative SRS seen off trial in *meta*-analyses compiling many retrospective cohorts [12]. LF may result from treatment deintensification with the dose de-escalation required to administer SRS to surgical cavities without creating an unacceptably high rate of RN[12]. For this reason, FSRT is garnering interest, as fractionation may facilitate safe delivery of a higher biological effective dose (BED) and improve LC[12]. A trend towards improved 1-year LC (86.8 % vs 68.0 %, p = 0.08) with postoperative FSRT compared to SRS was observed in a recent *meta*-analysis without any increase in RN (7 % vs 10 %, p = 0.46)[12]. Patients receiving postoperative FSRT in our study had a 9 % rate of symptomatic RN which is comparable to rates with postoperative FSRT presented in the *meta*-analysis by Lehrer et al [12]. To prospectively evaluate the potential benefit of FSRT, a randomized, phase III trial is currently underway (NCT04114981).

To improve outcomes, preoperative SRS has been proposed as an alternative to postoperative SRS. Preoperative SRS may reduce the risk of RN by using smaller target volumes with less irradiation of normal brain tissues, as postoperative cavities are larger than intact lesions[7], [13]. Pre-operative therapy may also eliminate the need to cover surgically manipulated tissues leading to a further reduction in treatment volume[7], [13]. Furthermore, preoperative SRS sterilizes the surgical field and thus may decrease the risk of MD from intraoperative spillage[7], [13]. To date, prospective evidence for pre-operative SRS is limited. A phase I dose escalation trial examining outcomes with preoperative SRS in 27 patients was presented at the American Society of Radiation Oncology (ASTRO) 2019 meeting and noted a 28 % rate of 1-year LF[14] which was similar to historical rates with postoperative SRS. Although the 1-year rate of MD was just 4 %, rates of RN rates were not documented. Additionally, an unpublished phase II single arm study was presented at ASTRO 2021 that noted a 1-year rate of LF of 10 % with preoperative SRS[15]. However, rates of RN and MD rates were not provided. In the retrospective setting, a multi-center cohort study noted 6 % and 15 % rates of 1-year MD and LF, respectively[7]. RN data was not provided. Additional single institution studies, some with mixed prospective and retrospective cohorts, have observed symptomatic RN, MD, and 1-year LF rates of 0–5 %, 0–17 %, and 14–50 % with pre-operative techniques [16], [17], [18], [19]. One retrospective analysis compared outcomes for preoperative versus postoperative SRS. The authors found that preoperative SRS resulted in decreased rates of 2-year symptomatic RN (5 % vs 16 %, p = 0.02) and MD (3 % vs 17 %, p = 0.01) without a difference in 2-year LF (23 % vs 16 %, p = 0.33)[13]. Our subset of patients who received preoperative SRS (n = 27) had a 7.4 % and 3.7 % rates of symptomatic grade 2 RN (without higher grade events) and MD, respectively, which were comparable to the 5 % and 3 % rates observed by (Patel 12 et al), and the rate of LF of just 3.7 % in this patient subset compares favorably to historical outcomes. In a limited sample size, the rate of LF, MD, and/or RN in our patients receiving postoperative SRS was 0 %, but this low rate is likely attributable to small sample size and selection bias favoring smaller tumors.

While these studies suggest that preoperative FSRT may offer improved rates of RN and MD without compromising cancer control, prospective outcomes with preoperative FSRT have not been previously reported. Mahajan et. al showed 0 %, 24 %, and 28 % rates of RN, LF, and MD yielding a composite endpoint of over 50 % with post-operative SRS[8]. On N107C, a seminal study establishing post-operative SRS as a standard treatment, there were 4 %, 38 %, and 7 % rates of Grade 2 or higher RN, LF, and MD in the SRS arm, resulting in a composite endpoint of up to 49 % with post-operative SRS arm [5]. On JCOG0504, there were a 3 %, 51 %, and 7 % rates of RN, LF, and MD yielding a composite endpoint of 61 % with postoperative SRS, although over 30 % of patients enrolled to receive SRS arm did not complete therapy as prescribed[6]. In comparison, the composite endpoint rate in the PROPS-BM cohort evaluating pre-operative SRS was 20.6 % and 24.8 % at one and two years, respectively[7]. In our study, the composite endpoint of 8 % for pre-operative FSRT compares favorably to prospectively evaluated post-operative SRS endpoints of 49–60 % and retrospectively evaluated pre-operative SRS endpoints of 20.6 %. It also compared favorably to our 15 % composite endpoint rate with preoperative SRS and 17 % rate with postoperative FSRT. The significantly decreased PTV volumes with preoperative FSRT may be responsible for numerically decreased rates of RN in our cohort, while the numerically decreased rates of LF and MD may be attributable to higher BED with fractionation and surgical sterilization, respectively. It is important to note that the use of a margin in all cases may also contribute to lower rates of LF in our cohort. Notably, PROPS-BM had more melanoma patients (12.8 % vs 7.5 %) and fewer gross total resections (93.7 % vs 98.1 %) then patients receiving FSRT on our study, both which were associated with LR in their analyses.

To our knowledge, this is the largest known study comparing pre-operative and post-operative FSRT. These data show that patients who receive pre-operative FSRT have good outcomes when compared to similar patient cohorts and compare favorable to previously published studies. Pre-operative treatment is more likely to be completed, especially with JCOG0504 showing that over 30 % of patients do not complete post-operative radiation treatment. Our composite endpoint has been previously published in other studies involving pre- and post-operative SRS. A major limitation of this study was the combination of small sample size and low event rate of LF, RN, and MD, which resulted in minimal power to detect differences between the treatment strategy groups. The use of a composite endpoint is imperfect given the differences in sequelae of the adverse outcomes. RN can be challenging to diagnose in both prospective and retrospective settings due to the lack of a standardized method of diagnosis. Another limitation of this study was that information comparing quality of life or neurocognition between cohorts was not available. Pre-operative radiation approach for a large or symptomatic brain metastasis may not be suited for all patients, particularly those with an uncertain diagnosis, for emergent situations, or for patients that are not stable on steroids. This may lead to selection bias when comparing treatment methods retrospectively. A large, randomized controlled trial is needed to compare outcomes between pre- and post-operative strategies.

## Conclusion

This study represents the largest comparison of outcomes with patients receiving pre-operative or post-operative FSRT. In our study, the use of pre-operative FSRT was safe without significant surgical morbidity, with all patients receiving planned radiation therapy. Pre-operative FSRT, when directly compared to single fraction and post-operative FSRT, had a clinically significant reduction of the composite endpoint from 15-17% to 8 %. Prospective validation of pre-operative FSRT is warranted.

## Declaration of Competing Interest

The authors declare that they have no known competing financial interests or personal relationships that could have appeared to influence the work reported in this paper.
